# Protective Effects of Selective β-Adrenoceptor Blockade on Renal Pathophysiology in a Catecholamine Storm of Rat

**DOI:** 10.3390/ijms27125480

**Published:** 2026-06-17

**Authors:** Bo-Hau Chen, Tzu-Hao Liu, Guan-Hong Lin, Hsin-Hung Chen, Yi-Ting Chu, Chih-Chieh Yang, Wen-Hsien Lu

**Affiliations:** 1 Department of Pediatrics, Taoyuan Armed Forces General Hospital, Taoyuan 325208, Taiwan; 2 Department of Pediatrics, Zuoying Armed Forces General Hospital, Kaohsiung 813204, Taiwan; 3 Department of Pediatrics, Kaohsiung Veterans General Hospital, Kaohsiung 813414, Taiwan; 4 Department of Medical Education and Research, Kaohsiung Veterans General Hospital, Kaohsiung 813414, Taiwan; 5 Department of Pediatrics, Pingtung Veterans General Hospital, Pingtung 900053, Taiwan; 6 Department of Health-Business Administration, Fooyin University, Kaohsiung 831301, Taiwan; 7 Institute of Biomedical Sciences, National Sun Yat-sen University, Kaohsiung 804201, Taiwan; 8 Department of Nursing, Shu-Zen Junior College of Medicine and Management, Kaohsiung 821004, Taiwan; 9 College of Health and Management, Cheng Shiu University, Kaohsiung 833301, Taiwan

**Keywords:** acute kidney injury, beta-adrenoceptor blocker, catecholamine storms, ferroptosis, metoprolol

## Abstract

Excessive administration of epinephrine and norepinephrine in critically ill patients may trigger a catecholamine storm and contribute to acute kidney injury (AKI) through activation of β-adrenoceptor signaling. Although clinical observations link high-dose catecholamine exposure to increased AKI risk, experimental models and mechanistic studies remain limited. We established a rodent model of combined epinephrine and norepinephrine infusion to investigate the renoprotective effects of subtype-selective β-adrenoceptor blockers. Animals received the β1-selective blockers metoprolol or atenolol, or the β2-selective blocker ICI 118,551. β1-adrenoceptor blockade, particularly with metoprolol, significantly attenuated renal histopathological injury and improved biochemical markers of kidney dysfunction. These protective effects were associated with suppression of ferroptosis-related pathways in the renal cortex. Atenolol partially improved biochemical parameters but did not significantly reduce tubulointerstitial damage, whereas β2-adrenoceptor blockade conferred limited functional benefit despite modest morphological improvement. Collectively, our findings indicate that β1-adrenoceptor activation plays a critical role in catecholamine-induced AKI by promoting ferroptosis. Targeting β1-adrenoceptors, especially with metoprolol, may represent a potential therapeutic strategy for preventing renal injury during catecholamine storms.

## 1. Introduction

Catecholamine storms, sometimes referred to as epinephrine (E) storms in several reports, are defined as a rapid surge of E and norepinephrine (NE) over a short period, leading to a multifactorial disorder that induces biochemical pathways responsible for cell damage [1]. Various pathophysiological or iatrogenic situations could raise catecholamine levels in circulation, leading to catecholamine storms. Tumor typessuch as pheochromocytoma and paragangliomaare able to secrete catecholamines in circulation, affecting the cardiovascular system directly, and induce several typical symptoms suchas uncontrolled paroxysmal hypertension, palpitations, pallor, perspiration and tachycardia [2]. Hypersensitivityof the sympatheticnerve from emotional stress or the fight-or-flight response triggers excessive E or NE secretion to induce several reversible pathological irregular disorders, such as Takotsubo cardiomyopathy [3]. Several severe pathogen infections, such as enterovirus 71, could induce catecholamine storms from sympatheticnerve hypersensitivity due to impairment of the nucleus of the solitary tract (NTS) in the medulla oblongata [4].

Numerous studies have shown that the rapid surge of circulating catecholamines caused by such high-dose injections can place significant stress on the cardiopulmonary system and may lead to damage in other organ systems [5,6]. In our previous study, continuous intravenous infusion of E + NE for6 hwas found to significantly reduce cardiac output and stroke work in animal models [7]. Our previous studies revealed that continuous intravenous infusion of E and NE significantly increased organ-system stress to a greater extent than infusion of E or NE alone in rodent models [7]. We also found that low-dose propranolol, a non-selective β-adrenoceptor blocker, could prevent catecholamine-induced acute heart failure and preserve cardiopulmonary function in two follow-up experiments [8,9]. Catecholamine storms also affect the lungs, with an overdose of E + NE leading to lung edema and pulmonary hypertension [7]. A recent study revealed that isoproterenol, a type of non-selective β-adrenoceptor agonist, can stimulate ferric ion accumulation in cardiomyocytes by upregulating the heme degradation pathway, thereby inducing ferroptosis [10]. In addition to cardiopulmonary injury, excess exogenous catecholamine administration may lead to other organ-systemic injuries due to microcirculatory blood flow redistribution. The studies from animal and clinical investigations revealed that exogenous E/NE administration could increase the risk of gastrointestinal damage with the retreat of blood flow from splanchnic circulation [6,11]. In the case of the kidneys, during a catecholamine storm, renal blood flow decreases rapidly due to vasoconstriction, limiting the supply of oxygen and nutrients. This leads to an accumulation of reactive oxygen species (ROS), which, in turn, causes renal tubular cell damage in the outer stripe of outer medulla (OSOM) and cortex, ultimately resulting in ischemic acute kidney injury (AKI) [12,13].

AKI is a risk factor affecting prognosis and survival rates in patients. Several clinical studies revealed that exogenous E/NE intervention in patients with emergency medical practice could increase the likelihood of AKI occurrence [5,14]. β-adrenoceptors play crucial roles in the renal pathophysiological response to E and NE. β1 and β2-adrenoceptors are two subtypes expressed in the kidneys [15]. β1-adrenoceptors are highly expressed in the juxtaglomerular apparatus cells, where they promote the renin–angiotensin II–aldosterone system. This leads to an increased rate of renin expression, which contributes to systemic hypertension through renal sympathetic nervous system hyperactivity, sodium and water retention, and upregulation of aquaporins in the collecting tubules [15,16,17,18]. In contrast, β2-adrenoceptors are primarily localized to the proximal tubule, mesangial cells of the glomeruli, and podocytes. They play a role in sodium elimination, limit Ca^2+^ influx into tubular cells, and participate in anti-inflammatory responses following pathogen invasion [15,19]. Although previous clinical studies have indicated that exogenous administration of E and NE increases the risk of AKI in resuscitated patients, regardless of whether the cause is cardiogenic [14], the molecular mechanisms underlying AKI induced by excessive catecholamine exposure and potential protective strategies remain insufficiently studied.

Although clinical observations have suggested that excessive epinephrine administration during resuscitation may increase the risk of AKI, there are currently no basic research studies reporting animal models of catecholamine-induced AKI [14]. In our previous work, we demonstrated that β-adrenoceptor blockers exert protective effects against cardiac injury caused by excessive catecholamines. Based on these findings, we hypothesize that β-adrenoceptor blockers may also have protective potential against catecholamine-induced AKI. Using our well-established rat model with exogenous administration of E and NE, we aim to establish a mechanistic link between high-dose E + NE administration and AKI and to investigate the therapeutic efficacy and protective potential of different β-adrenoceptor blockers in this setting.

## 2. Results

### 2.1. Metoprolol and ICI 118,551 Alleviates Severe Tubular Damage in the Renal Cortex and OSOM Induced by Catecholamine Excess

**Figure 1:**
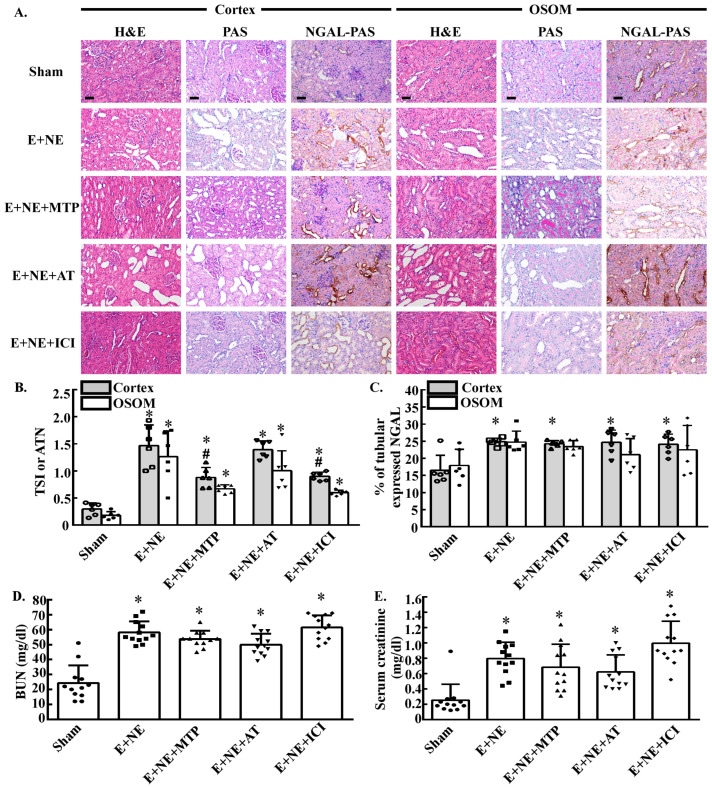
Histological changes and injury levels in renal tubulointerstitial tissue of cortex and OSOM after administration of β-adrenoceptor blockers in E + NE models. (**A**) The morphology of the renal tubulointerstitial region in the cortex and the OSOM was assessed using H&E staining. Pathological features were observed with PAS. NGAL expression was evaluated using IHC-PAS co-staining. All images were captured at 200× magnification, with a scale bar of 50 μm. (**B**) Tubulointerstitial lesion scores in the cortex and acute tubular necrosis scores were quantified based on PAS staining. Sample size: *n* = 6 for all groups. (**C**) The percentage of NGAL-positive tubules was quantified based on NGAL IHC-PAS co-staining. Sample size: *n* = 6 for all groups. (**D**,**E**) Serum biochemical parameters of renal function, including BUN (**D**) and creatinine (**E**), were measured. Data are presented as means ± SD. Statistical analyses were performed using the Kruskal–Wallis H test and post hoc Mann–Whitney U test.*, *p* < 0.05 vs. sham; #, *p* < 0.05 vs. E + NE. Sample size: *n* = 12 for all groups. In the statistical figures, circles represent the sham group, squares represent the E + NE group, triangles represent the E + NE + MTP group, inverted triangles represent the E + NE + AT group, and diamonds represent the E + NE + ICI group. AT: atenolol, BUN, blood urea nitrogen; E, epinephrine; H&E, Hematoxylin and Eosin staining; ICI, ICI 118,551; IHC, immunohistochemistry; MTP, metoprolol; NE, norepinephrine; NGAL, neutrophil gelatinase-associated lipocalin; OSOM, outer stripe of the outer medulla; PAS, Periodic Acid–Schiff staining.

To confirm the occurrence of kidney injury induced by excessive catecholamines and to evaluate the efficacy of different β-adrenoceptor blockers, we examined histological alterations and biochemical markers associated with renal damage. Histological analysis revealed that tubular necrosis and dilation were prominent in the renal cortex and OSOM in E + NE rodent models, as observed using hematoxylin and eosin staining (Figure 1A). Additionally, PAS staining demonstrated the loss of the brush border in injured tubules and the thyroidization of tubules (Figure 1A). Morphological injuries in the renal cortex and OSOM were significantly more severe in the E + NE groups compared to the sham group. E + NE + MTP or E + NE + ICI groups significantly alleviated the E + NE-induced morphological damage in both the cortex and OSOM. However, no significant differences in morphological injury were observed between the E + NE + AT groups and the sham group (Figure 1A). When evaluating the TSI of the cortex and the ATN of the OSOM based on PAS staining, injury scores in the E + NE group were higher than those in the sham group. Intervention with MTP effectively reduced histological injury caused by catecholamine overload, whereas AT did not produce significant improvements. Interestingly, tubular damage levels were also alleviated in the E + NE + ICI groups, contrary to expectations (Figure 1B). The expression of neutrophil gelatinase-associated lipocalin (NGAL) was detected using IHC staining. To facilitate the observation of tubular morphology, PAS staining was used as a counterstain to highlight the renal parenchyma. In the sham group, NGAL expression was confined to the distal tubules in the cortex and the ascending tubules in the OSOM. However, in catecholamine-induced kidney injury, NGAL expression appeared in injured proximal tubules of the cortex and descending tubules of the OSOM. Notably, some severely dilated and necrotic tubules did not express NGAL molecules (Figure 1A). NGAL expression was markedly increased in the renal cortex of E + NE-infused groups, regardless of β-adrenoceptor blocker intervention. However, when compared to the E + NE group, no significant differences in NGAL expression levels were observed with either β1- or β2-adrenoceptor blocker administration. Similarly, NGAL expression levels in the OSOM showed no significant changes but followed a trend consistent with the data observed in the cortex (Figure 1C). Although vessel lesions observed by PAS staining increased in some individuals following E + NE infusion in the arcuate arteries between the renal cortex and OSOM, no statistically significant differences were detected among the groups (Figure S1A,B). Quantitative analysis of lumen and vessel wall space using EVG revealed a significant narrowing of the arcuate artery lumens in the E + NE groups compared to the sham group. Notably, β-adrenoceptor blockers, particularly ICI, demonstrated the ability to dilate these arteries (Figure S1A,C). BUN and creatinine levels were markedly elevated in E + NE-infused groups (Figure 1D,E). Intervention with β1-adrenoceptor blockers, particularly AT, restored most biochemical markers, whereas renal injury markers were more pronounced in the β2-adrenoceptor-blocker-treated E + NE groups (Figure 1D,E).

### 2.2. The Alterations in β-Adrenoceptors Are Observed Following a Catecholamine Storm, Either Alone or in Combination with Various β-Blocker Treatments in Kidney Tissue

**Figure 2:**
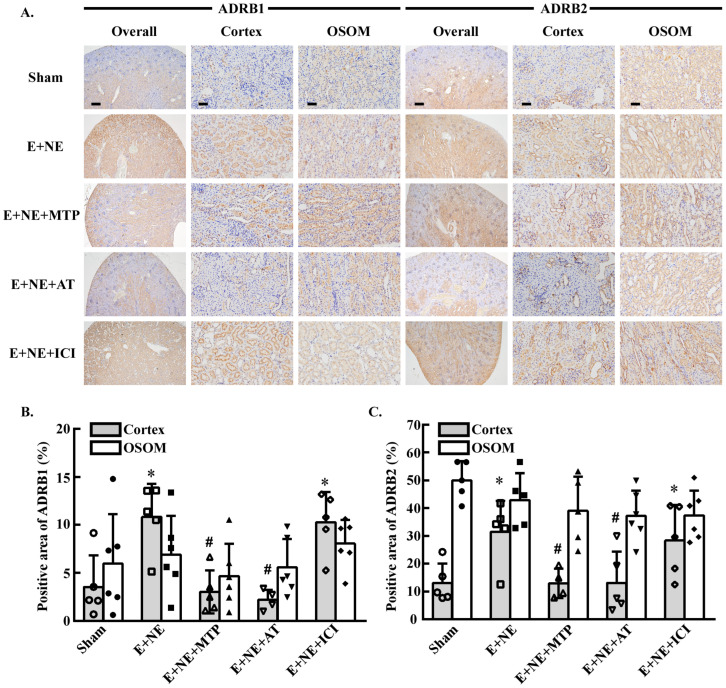
Expression levels of β1- and β2-adrenoceptors following β-adrenoceptor blocker interventions in E + NE catecholamine storm models. (**A**) The distribution and expression levels of ADRB1 and ADRB2 were visualized with IHC staining. Low-magnification images (40×, scale bar: 20 μm) depicted the overall distribution of ADRB1 and ADRB2 across the kidney tissue. High-magnification images (200×, scale bar: 50 μm) provided detailed visualization of ADRB1 and ADRB2 expression specifically in the renal cortex and the OSOM. (**B**,**C**) The fraction of ADRB1 (**B**) and ADRB2 (**C**) crossing the cortical and OSOM sections was measured to estimate the impact of different β-adrenoceptor blockers on iron distribution in these regions. Data are presented as means ± SD. Statistical analyses were performed using the Kruskal–Wallis H test and post hoc Mann–Whitney U test. *, *p* < 0.05 vs. sham; *#*, *p* < 0.05 vs. E + NE. Sample sizes: *n* = 5 for all groups in cortex and *n* = 6 for all groups in OSOM (**B**); sham (*n* = 5), E + NE (*n* = 5), E + NE + AT (*n* = 4), E + NE + MTP (*n* = 5), E + NE + ICI (*n* = 5) in cortex and sham (*n* = 5), E + NE (*n* = 5), E + NE + AT (*n* = 5), E + NE + MTP (*n* = 6), E + NE + ICI (*n* = 6) in OSOM (**C**).In the statistical figures, circles represent the sham group, squares represent the E + NE group, triangles represent the E + NE + MTP group, inverted triangles represent the E + NE + AT group, and diamonds represent the E + NE + ICI group. ADRB1, β1-adrenoceptor; ADRB2, β2-adrenoceptor; AT, atenolol; E, epinephrine; ICI, ICI 118,551; IHC, immunohistochemistry; MTP, metoprolol; NE, norepinephrine; OSOM, outer stripe of the outer medulla.

As key sensors for detecting E and/or NE, adrenoceptors play a critical role in catecholamine storms by initiating downstream signaling cascades. We employed immunohistochemistry (IHC) to assess the expression levels and localization of β1- and β2-adrenoceptors in kidney tissues from the E + NE model in order to evaluate the effects of β-adrenoceptor blocker intervention. The expression of both β1-adrenoceptor (ADRB1) and β2-adrenoceptor (ADRB2) increased in the apical region of tubular epithelial cells following catecholamine infusion (Figure 2A). ADRB1 expression was significantly reduced in the E + NE + AT andE + NE + MTP groups (Figure 2B). In contrast, no significant difference in ADRB1 expression was observed in the E + NE + ICI group compared to the E + NE group (Figure 2B). In the E + NE + AT and E + NE + MTP groups, both ADRB1 and ADRB2 expression levels were suppressed (Figure 2B,C). Interestingly, ADRB2 expression remained unchanged and was not restored in the E + NE + ICI groups (Figure 2C).

### 2.3. β-Adrenoceptor Blockade Mitigates Oxidative DNA Damage, Apoptosis, and Iron Accumulation in E + NE-Induced Renal Injury

**Figure 3:**
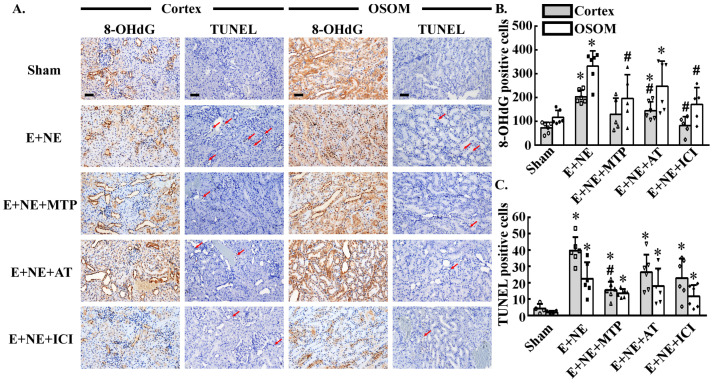
Nuclear DNA damage in renal tubular cells of the cortex and OSOM following β-adrenoceptor blocker treatment in a catecholamine-overload rat model. (**A**) Representative images of oxidative DNA damage marker 8-OHdG and TUNEL staining in renal tissue sections. Images were acquired at 200× magnification; scale bar = 50 μm. (**B**) Quantification of 8-OHdG-positive nuclei to assess oxidative DNA damage. Sample sizes: sham (*n* = 6), E + NE (*n* = 6), E + NE + AT (*n* = 5), E + NE + MTP (*n* = 6), E + NE + ICI (*n* = 5). (**C**) Quantification of TUNEL-positive nuclei to evaluate DNA fragmentation. Sample sizes: sham (*n* = 4), E + NE (*n* = 6), E + NE + AT (*n =* 6), E + NE + MTP (*n* = 6), E + NE + ICI (*n* = 6).In the statistical figures, circles represent the sham group, squares represent the E + NE group, triangles represent the E + NE + MTP group, inverted triangles represent the E + NE + AT group, and diamonds represent the E + NE + ICI group. Data are expressed as mean ± SD. Statistical analyses were performed using the Kruskal–Wallis H test followed by Mann–Whitney U post hoc comparisons. * *p* < 0.05 vs. sham; # *p* < 0.05 vs. E + NE. Abbreviations: 8-OHdG, 8-hydroxy-2′-deoxyguanosine; AT, atenolol; E, epinephrine; ICI, ICI 118,551; MTP, metoprolol; NE, norepinephrine; OSOM, outer stripe of the outer medulla; TUNEL, terminal deoxynucleotidyl transferase dUTP nick-end labeling.

Immunohistochemical analysis of 8-OHdG, a marker of oxidative DNA damage, revealed a significant increase in the number of 8-OHdG-positive nuclei in both the renal cortex and OSOM in the E + NE group compared to the sham control. In addition, most of the numbers of 8-OHdG-positive cells in the cortex or the OSOM were significantly reduced when either the β1-or the β2-adrenoceptor blocker intervened in E + NE-infused rats (Figure 3A,B). Quantitative analysis of TUNEL staining demonstrated a significant increase in TUNEL-positive cells (red arrows) in the kidneys of rats subjected to excessive E + NE infusion. Intervention with β-adrenoceptor blockers resulted in a decreasing trend in TUNEL-positive cell numbers, with a statistically significant reduction observed in the cortical region of the E + NE + MTP group (Figure 3A,C).

**Figure 4:**
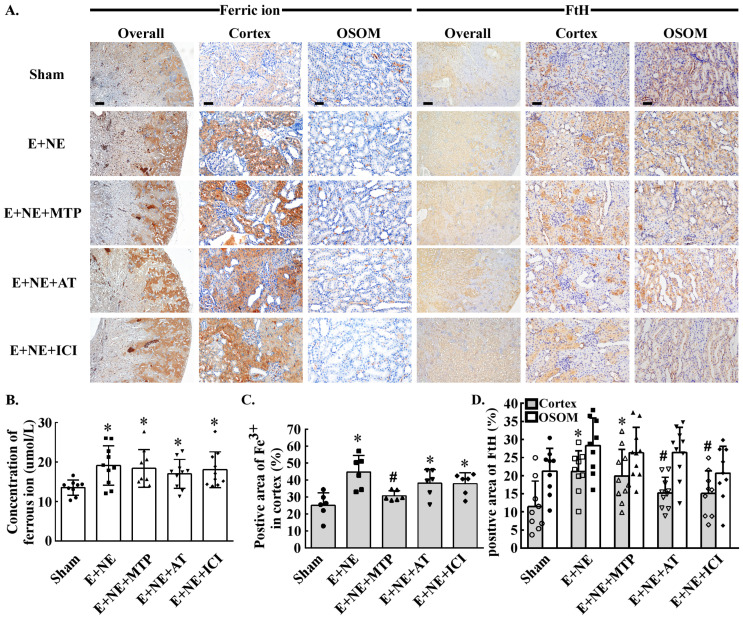
Effect of different β-adrenoceptor blockers on the distribution of iron ions. (**A**) The distribution of iron and FtHwasobserved in the overall kidney at low magnification (40×) or in the cortex or OSOM athigh magnification (200×). Scale bars: 20 μm (40× field) and 50 μm (200× field). (**B**) The concentration of ferrous ions (Fe^2+^) in serum was measured using ELISA. Sample sizes: sham (*n* = 9), E + NE (*n* = 10), E + NE + AT (*n* = 9), E + NE + MTP (*n* = 10), E + NE + ICI (*n* = 10). (**C**) The fraction of ferric ions (Fe^3+^) crossing the cortical section was quantified with Prussian blue staining. (**D**) The fraction of FtH crossing the cortical and OSOM sections was measured with IHC staining. Sample sizes:sham (*n* = 9), E + NE (*n* = 10), E + NE + MTP (*n* = 10), E + NE + AT (*n* = 11), E + NE + ICI (*n* = 9). In the statistical figures, circles represent the sham group, squares represent the E + NE group, triangles represent the E + NE + MTP group, inverted triangles represent the E + NE + AT group, and diamonds represent the E + NE + ICI group. Data are presented as means ± SD. Statistical analyses were performed using the Kruskal–Wallis H test and post hoc Mann–Whitney U test.*, *p* < 0.05 vs. sham; #, *p* < 0.05 vs. E + NE. AT, atenolol; E, epinephrine; ELISA, enzyme-linked immunosorbent assay; FtH, ferritin heavy chain; ICI, ICI 118,551; IHC, immunohistochemistry; MTP, metoprolol; NE, norepinephrine; OSOM: outer stripe of the outer medulla.

The storage and release of free iron ions within cells are critical factors in regulating iron homeostasis and the ferroptosis pathway. Both the expression levels and the spatial distribution of iron-related molecules must be considered to understand the role of iron in ferroptosis across different regions of kidney tissue. The localization of ferritin heavy chains (FtH) and ferric ions in kidney tissues showed distinct patterns. Under low magnification, ferric ions were predominantly expressed in the renal cortex, whereas FtH proteins were primarily localized to the OSOM (Figure 4A). The expression area fractions of both ferritin and ferric ions were significantly higher in E + NE-treated groups compared to the sham group. The serum concentration of ferrous ions increased significantly during catecholamine storms. However, administration of β-adrenoceptor blockers in E + NE models did not effectively reduce ferrous ion levels (Figure 4B). Interestingly, the administration of either β1- or β2-adrenoceptor blockers often resulted in opposing trends for these markers (Figure 4C,D). Ferric ions were rarely detected in the tubules of the OSOM across all groups (Figure 4A). Similarly, the expression area fraction of FtH proteins in the OSOM did not show significant changes among the groups (Figure 4A,D).

### 2.4. β1-Adrenoceptor Blockade Improves Injury, Ferroptosis and Oxidative Stress-Related Protein Levels in Kidneys Under Catecholamine Excess

**Figure 5:**
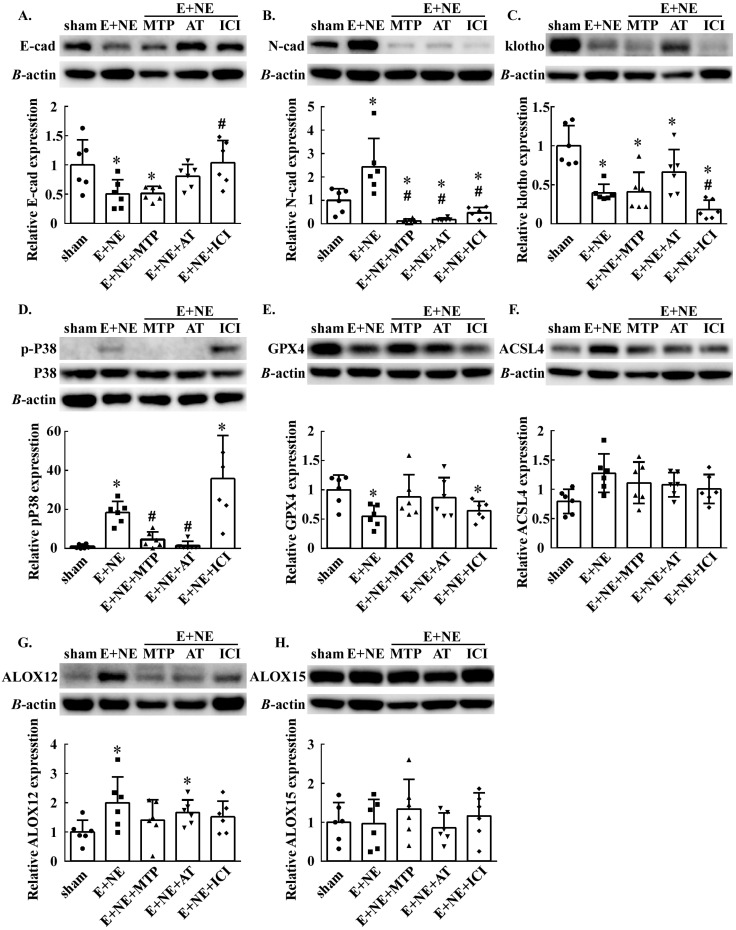
Molecular expression analysis of catecholamine-induced acute kidney injury in kidney tissue. (**A**–**H**) Immunoblotting was used to analyze the protein expression levels ofE-cadherin (**A**), N-cadherin (**B**), Klotho (**C**), total p38 and phosphorylated p38 (p-p38) (**D**), GPX4 (**E**), ACSL4 (**F**), ALOX12 (**G**), and ALOX15 (**H**) in kidney tissue. Data are presented as means ± SD. *n* = 6 for all groups. In the statistical figures, circles represent the sham group, squares represent the E + NE group, triangles represent the E + NE + MTP group, inverted triangles represent the E + NE + AT group, and diamonds represent the E + NE + ICI group. Statistical analyses were performed using the Kruskal–Wallis H test and post hoc Mann–Whitney U test. *, *p* < 0.05 vs. sham; #, *p* < 0.05 vs. E + NE. ACSL4, acyl-CoA synthetase long chain family member 4; ALOX12, arachidonate 12-lipoxygenase; ALOX15, arachidonate 15-lipoxygenase; AT, atenolol; E: epinephrine; GPX4, glutathione peroxidase type 4; ICI, ICI 118,551; MTP, metoprolol; NE, norepinephrine.

We observed that excessive catecholamine exposure induced renal morphological alterations and iron accumulation in kidney tissue, which were modulated by the administration of different β-adrenoceptor blockers. Based on these findings, we further investigated molecular changes associated with epithelial injury and ferroptosis. In particular, epithelial–mesenchymal transition and profibrotic responses are key processes used to assess renal tubular injury. E-cadherin (E-cad) and N-cadherin (N-cad) serve as markers for tubular epithelial differentiation and injury [20]. During epithelial–mesenchymal transition in injured kidney tubules, the expression of E-cad decreases, while that of N-cad increases. We reproduced this expression pattern, confirming that tubular injury occurs following excessive catecholamine infusion (Figure 4A,B). Most β-adrenoceptor blockers used in the E + NE models reversed these changes in E-cad and N-cad expression (Figure 5A,B). The anti-fibrotic marker klotho was reduced in E + NE models. While E + NE + MTP or E + NE + AT groups did not significantly alter klotho expression compared to E + NE models, E + NE + ICI groups further decreased klotho expression (Figure 5C). Phosphorylation of p38 MAPK is an important marker of MAPK pathway activation, which contributes to the accumulation of ferric ions in the cytoplasm and is triggered by β-adrenoceptor activation [21,22,23]. In E + NE + MTP or E + NE + AT groups, the phosphorylation level of p38 was significantly lower than in the E + NE group without β-adrenoceptor blockade (Figure 5D). The expression level of glutathione peroxidase 4 (GPX4), a critical enzyme involved in the glutathione pathway for antioxidative defense, was significantly reduced in the E + NE groups compared to the sham group. E + NE + MTP or E + NE + AT groups restored GPX4 expression to levels comparable to the sham group, although this recovery was not statistically significant when compared to the E + NE group (Figure 5E). In contrast, neither p38 phosphorylation nor GPX4 expression recovered in the E + NE + ICI groups (Figure 5D,E). The peroxidation of polyunsaturated fatty acids (PUFAs) is a critical process that induces cellular damage following activation of the ferroptosis pathway. This lipid peroxidation involves the activation of fatty acid chains through CoA conjugation and subsequent oxidation of PUFAs. The expression of Acyl-CoA Synthetase Long-Chain Family Member 4 (ACSL4), a key enzyme involved in the synthesis of polyunsaturated fatty acids through CoA conjugation to long-chain fatty acids, was significantly elevated in catecholamine-treated rats compared to the sham group (Figure 5F). Although no significant intergroup differences were observed, the trend of ACSL4 expression paralleled that of the FtH area fraction. Arachidonic acid peroxidation is a common pathway for generating lipid peroxides during ferroptosis, catalyzed by arachidonate lipoxygenase (ALOX) enzymes.We observed an increase in the expression of arachidonate 12-lipoxygenase (ALOX12), but not arachidonate 15-lipoxygenase (ALOX15), in the renal tissue of E + NE rodent models (Figure 5G,H). Administration of either β1- or β2-adrenoceptor blockers in E + NE rodent models appeared to partially restore ALOX12 expression toward levels seen in the sham group. However, this reduction was not statistically significant (Figure 5G).

## 3. Discussion

Our data suggest that β1-adrenoceptor blockers can effectively reduce catecholamine-induced renal injury, potentially in association with modulation of ferroptosis-related pathways. The animal model we developed accurately represents the clinical features of AKI induced by high doses of catecholaminergic agents. We observed that the trends of profibrotic molecules, p38 phosphorylation, and GPX4 expression corresponded with renal function changes. By examining two distinct histological layers—the cortex and the OSOM—we innovatively demonstrated the effects of different β-adrenoceptor blockers in catecholamine-induced AKI. Notably, although both MTP and AT improved renal function, only MTP provided protection against pathological tubular damage caused by catecholamine overdose. Interestingly, tubular injuries in the kidney tissue of the E + NE + ICI group showed contradictory results when compared with renal injury markers. Ferric ions and ferritin exhibited strong localized expression in different kidney layers, with MTP uniquely reducing ferric ion accumulation in the renal cortex of E + NE models. In summary, our findings suggest that β1-adrenoceptors are involved in catecholamine-induced renal injury, with concurrent alterations observed in ferroptosis-related pathways. MTP may therefore hold therapeutic potential for attenuating AKI under these conditions.

Excess catecholamines can occur during emergency treatment for critically ill patients receiving high doses of adrenaline, in cases of pheochromocytoma, or under extreme emotional stress [24]. This condition can lead to multi-organ damage, including cardiomyopathy, acute kidney disease (AKI), lung injury, and immune hyperactivation [10,25,26,27,28]. In the kidneys, excessive doses of adrenaline and/or noradrenaline promote renal vasoconstriction, resulting in hypovolemia and hypoperfusion, which can ultimately cause ischemic ATN [29]. Clinical research has shown that high-dose E administration improves resuscitation rates in patients with cardiac arrest compared to standard doses [25,30]. However, some clinical cohort studies have reported that high-dose E increases the incidence of AKI in patients after a returntospontaneouscirculation, paradoxically reducing post-resuscitation survival rates [25,31]. Although previous studies indicate that continuous high-dose catecholamine treatment increases the risk of AKI in clinical settings, the underlying mechanisms remain poorly understood. Our E + NE rodent model provides an innovative and reliable reflection of the kidney damage caused by excessive catecholamines, effectively mirroring clinical phenomena.

The mechanism of β-adrenoceptors plays a critical role in the catecholamine signaling transduction cascade, regulating molecular expression and physiological activation. While low-dose propranolol, a non-selective β-adrenoceptor blocker, has been shown to reverse catecholamine-induced damage, the specific functions of individual β-adrenoceptor subtypes in organ-system injuries remain unclear [9]. To address this, we utilized selective β-adrenoceptor blockers to target specific subtypes. AT and MTP, both selective β1-adrenoceptor blockers, are widely used in clinical therapies. Despite their similar chemical structure and reactive targets, AT and MTP exhibit distinct pharmacological and physiological features [32]. MTP, with an alkoxy group at the para-position of its benzyl structure, is more lipophilic and metabolically active in the liver compared to AT [32]. It undergoes a P450-dependent metabolic process in hepatocytes, primarily involving enzymes such as CYP2D6, which transforms it into less active forms, resulting in a shorter half-life than AT [33,34]. In contrast, AT is relatively hydrophilic, does not undergo significant metabolic transformation, and retains its full activity until excretion [34]. Although propranolol shares a similar metabolic pathway with MTP, its metabolites retain high β-adrenoceptor-blocking activity [34,35]. Early reports indicate that the dosage of β-adrenoceptor blockers should be reduced in patients with renal disease, as the active drugs or their metabolites may accumulate during excretion, prolonging their effects [34]. Even though clinical case reports have revealed that AT has the potential to induce AKI in patients with chronic kidney disease, further investigations into the underlying mechanisms have not been conducted or elucidated [36]. A previous study indicated that AT, similar to certain organic drugs such as metformin, is eliminated from the body via organic cation transporter 2 (OCT2) and multidrug and toxin extrusion protein (MATE), which are expressed in the S2 segment of the renal cortex and the S3 segment of the OSOM [37]. AT is transported from the bloodstream into tubular epithelial cells by OCT2 and subsequently excreted into the tubular lumen via MATE [38,39]. This elimination pathway enables AT to be absorbed into tubular cells. However, no study to date has clarified whether AT transported into tubular epithelial cells via the OCT2/MATE system can interfere with intracellular signaling pathways in the cytosol and thereby exacerbate tubulointerstitial injury in the context of renal dysfunction. We believe that investigating the intracellular effects of AT in tubular epithelial cells will help to bridge this current gap in knowledge. In the present study, although both agents are β1-adrenoceptor blockers, MTP demonstrated greater efficacy than AT in attenuating catecholamine-induced injury at some histological levels. While this difference may be partially attributable to their distinct metabolic profiles, direct evidence supporting the superiority of MTP over AT in this context remains limited. Therefore, further studies are warranted to elucidate the underlying molecular mechanisms responsible for their differential effects in catecholamine-induced AKI, which may provide valuable insights with translational and clinical relevance.

β1- and β2-adrenoceptors exhibit distinct cell signaling responses, leading to different physiological effects [40,41,42]. Both subtypes are associated with G-protein–adenylyl-cyclase–cAMP pathways, but their specific interactions differ. For β1-adrenoceptors, G_s_ proteins primarily enhance adenylyl cyclase (AC) activity, whereas β2-adrenoceptors can interact with both G_s_ and G_i_ proteins, resulting in either stimulatory or inhibitory effects on downstream AC signaling [40]. Recent studies reveal that β2-adrenoceptors can also activate β-arrestin-mediated signaling pathways independent of G-proteins when internalized into endosomes [41]. The G_s_-to-G_i_ protein trafficking mechanism in β2-adrenoceptors plays a critical role in their functional modulation, particularly under conditions of E overload and β1-adrenoceptor hyperactivity. At high E concentrations, the coupling of β2-adrenoceptors shifts from G_s_ to G_i_, antagonizing β1-adrenoceptor activity at the signaling transduction level [42]. Interestingly, NE overload does not trigger this G_s_-to-G_i_ shift [42]. In vascular endothelial cells, β2-adrenoceptors coupled to G_s_ proteins initiate a positive feedback loop of catecholamine synthesis via the AC–cAMP–PKA signaling pathway [43]. Protein kinase A (PKA), once activated, phosphorylates downstream targets, including β2-adrenoceptors themselves. This phosphorylation leads to a switch in the associated G protein from G_s_ to G_i_, altering the downstream signaling cascade [43]. Local distribution and expression levels of β2-adrenoceptors also influence whether G_s_-G_i_ trafficking occurs. ICI 118,551, a β2-adrenoceptor inverse agonist, stabilizes the receptor’s inactive form, thereby inhibiting the AC-cAMP cascade [42]. It enhances G_i_ activity while inhibiting G_s_ activity in β2-adrenoceptors. Previous studies also indicated that G_i_ proteins play a critical role in the inhibitory action of ICI 118,551 on β2-adrenoceptor signaling [44,45]. The suppressive effect of ICI 118,551 was abolished when G_i_ proteins were inhibited by pertussis toxin (PTX) [44,45]. Recent evidence suggests that β_2_-adrenoceptors can modulate nitric oxide (NO)-mediated vasodilation through G_i/o_ protein coupling [45]. The β_2_-adrenoceptor inverse agonist ICI 118,551 has been shown to stabilize the β_2_AR–G_i_ complex, thereby inhibiting the downstream AC–PKA pathway [42]. Prior studies have demonstrated that ICI 118,551 exerts vessel-specific effects on vasoreactivity, attenuating norepinephrine-induced vasoconstriction in pulmonary arteries but not in the aorta [45]. These findings highlight the heterogeneity of β_2_-adrenoceptor-mediated NO signaling across different vascular beds. However, whether a similar mechanism contributes to the regulation of renal arcuate artery tone under conditions of excessive catecholamine exposure remains unexplored. In addition to β1- and β2-adrenoceptors, β3-adrenoceptors have emerged as potential regulators of renal function [46]. These receptors are primarily located in the renal medulla, where they activate the AC–cAMP pathway to promote water and sodium reabsorption by regulating the expression of aquaporin 2 (AQP2) and Na–K–Cl cotransporter type 2 (NKCC2) [46]. However, the underlying mechanisms of β3-adrenoceptor-mediated regulation in kidney metabolism remain poorly understood. Our results show that the E + NE + ICI group exhibited more severe renal injury, as indicated by biochemical parameters, compared to the E + NE group (Figure 1D,E). ADRB2 expression was higher in the E + NE + ICI group than in other groups, and ADRB1 expression appeared to be compensatorily upregulated (Figure 2B). These findings suggest that ADRB2 cooperates with G_i_ to inhibit the AC-PKA pathway indirectly, while ICI 118,551 promotes the AC-PKA pathway by maintaining the inactive form of ADRB2 and upregulating ADRB1 expression. Currently, no research has focused on the relationship between β2-adrenoceptor intervention in catecholamine storms and histological changes in kidney tissue. Our study provides innovative insights, showing that tubulointerstitial damage in the E + NE + ICI group was significantly attenuated compared to the E + NE group, contradicting other findings. This result highlights the complexity of β2-adrenoceptor blocker involvement in catecholamine-induced AKI. Therefore, the clinical utility of β2-adrenoceptor blockers in treating catecholamine-induced AKI may be limited, suggesting additional mechanisms involving β2-adrenoceptors in this condition.

Ferroptosis, a newly identified form of nonapoptotic cell death dependent on ferric ions, contributes to cellular damage through lipid peroxidation, polyunsaturated fatty acid formation, iron transport, and antioxidant production [47,48,49,50,51,52]. While studies have linked the ferroptosis pathway to cardiotoxicity in catecholamine-overloaded rodent models, its role in tubulointerstitial injury during catecholamine-induced AKI remains unclear [10]. Before the concept of ferroptosis was proposed, clinical reports had already described the use of iron chelators in the treatment of AKI [53]. In addition, numerous experimental studies have reported the therapeutic application of iron chelators in AKI induced by various methods [54,55,56,57]. While these clinical and preclinical studies have demonstrated the protective effects of iron chelation in AKI triggered by diverse insults, no prior research has addressed catecholamine-induced AKI in this context. Specifically, the therapeutic potential of iron chelators, the role of ferroptosis, and the mechanistic link between β-adrenergic receptor signaling and ferroptosis in catecholamine-induced AKI have not been previously explored. A phenomenon was observed in the E + NE + MTP group, where a reduction in intracellular iron was accompanied by amelioration of renal injury (Figure 1B and Figure 4C). Our findings confirm elevated serum ferrous ions in E + NE groups, with no reduction after β-adrenoceptor blockade, suggesting additional mechanisms in ferric ions transferred across cell membranes (Figure 4B). A recent article also revealed that ferric ions accumulated in the cell from catecholamine overdose due to heme degradation rather than increased total body iron [10]. Our investigations not only aligned with the previous report but also further supplemented the results obtained in this study. Ferric ions and FtH exhibited distinct distributions in kidney tissue, with ferritins most abundant in the OSOM, while significant ferric metabolism changes occurred in the cortex (Figure 4A). Additionally, lipid peroxidation was regulated by ferric ion catalysis and the system xc^-^ pathway, with ferric ions increasing ROS through the Fenton reaction and detoxification by glutathione [58]. Compared to E + NE groups, there is atrend of restoring ferroptosis-related molecules to the same level as the sham groups with β1-adrenoceptor blocker treatment (Figure 5D–G). These improvements in kidney damage and renal function suggest that MTP may be a potential candidate for partially preventing renal ferroptosis during catecholamine-induced AKI.

Phosphorylation of p38 MAPK appears to be a critical molecular event linking β-adrenoceptor signaling to the ferroptosis pathway [43,59]. Upon activation of β-adrenoceptors coupled to G_s_ proteins, the downstream AC–cAMP–PKA signaling cascade is triggered, leading to the phosphorylation of p38 MAPK, which in turn promotes pro-inflammatory responses and ferroptotic cell death [59]. A previous study demonstrated that p38 MAPK phosphorylation can be stimulated by isoproterenol, a dual β1/β2-adrenoceptor agonist, and by forskolin, a direct cAMP activator, whereas inhibition of PKA significantly suppressed this phosphorylation event [60]. Importantly, the same study reported that inhibition of G_i_ proteins using pertussis toxin (PTX) did not reduce p38 MAPK phosphorylation in isoproterenol-treated cardiomyocytes, indicating that the activation of p38 MAPK is primarily mediated by G_s_ protein signaling rather than G_i_ pathways [60]. Our findings are consistent with these observations. In the E + NE-treated group, phosphorylation of p38 MAPK was markedly increased, supporting the involvement of G_s_-protein-coupled β-adrenoceptors in this signaling cascade. Administration of β1-adrenoceptor blockers, including MTP and AT, significantly attenuated p38 MAPK phosphorylation (Figure 4D), further supporting the role of β1-adrenoceptor–G_s_–PKA signaling in activating this pathway. Notably, in the E + NE + ICI group, p38 MAPK phosphorylation not only failed to return to sham levels but was even higher than in the untreated E + NE group (Figure 4D). This unexpected elevation may be explained by the compensatory upregulation of ADRB1 observed in the E + NE + ICI group (Figure 2B). Since ICI selectively inhibits β2-adrenoceptors, its administration likely disrupts β2-mediated signaling and shifts the balance toward increased β1-adrenoceptor activity, thereby enhancing G_s_-mediated p38 MAPK phosphorylation. These results further support the notion that p38 MAPK activation is predominantly regulated by β1-adrenoceptor–G_s_–PKA signaling during catecholamine-induced kidney injury.

## 4. Materials and Methods

### 4.1. Experimental Animals

Sprague-Dawley rats (RRID:RGD_70508) were purchased from the Yilan Breeding Center, BioLasco Taiwan Co., Ltd. (Yilan County, Taiwan) and housed at the Experimental Animal Center, Kaohsiung Veterans General Hospital (Kaohsiung City, Taiwan). Animals were provided with food and water ad libitum, and environmental conditions, including temperature and humidity, were maintained according to IACUC regulations. A 12 h light/dark cycle was strictly followed. All animals were acclimated to the Experimental Animal and Surgical Training Center for at least one week before the start of the experiments. All animal research procedures were reviewed and approved by the Institutional Animal Care and Use Committee of Kaohsiung Veterans General Hospital.

### 4.2. Epinephrine and Norepinephrine Infusion with or Without Beta-Blocker Bolus

Male rats (7 to 9 weeks old) weighing 320–380 g were anesthetized via intraperitoneal administration of urethane (1 g/kg). Femoral vein catheterization was performed using PE50 tubing. E (4.5 μg/kg/min) (Taiwan Biotech Co., Taoyuan, Taiwan) and NE (6.8 μg/kg/min) (Tai Yu Chemical & Pharmaceutical Co., Hsinchu, Taiwan) were continuously administered via the left femoral vein at a steady rate for 6 h [9]. Metoprolol (MTP, 2 mg/kg) (Merck KGaA, Darmstadt, Germany; M5391) [61,62], a β1-adrenoceptor blocker, was dissolved and diluted in physiological saline, while atenolol (AT, 2 mg/kg) (Merck KGaA, Darmstadt, Germany; A7655) [63], another β1-adrenoceptor blocker, and ICI 118,551 (ICI, 0.5 mg/kg) (Merck KGaA, Darmstadt, Germany; I127) [64], a β2-adrenoceptor blocker, were dissolved in dimethyl sulfoxide (99.5% purity) and subsequently diluted with physiological saline. The final concentration of dimethyl sulfoxide after dilution with physiological saline was maintained at 18% *v*/*v*. These β1- and β2-adrenoceptor blockers, respectively, were administered as a bolus injection after 1 h of continuous E + NE infusion. Rats in the sham group received continuous infusion of physiological saline for 6 h.Following the infusion period, the animals were euthanized via cardiac puncture, and the kidneys were harvested for further analysis. The harvested kidneys were fixed in 10% neutral phosphate-buffered formalin for 5 days, dehydrated in a graded alcohol series, cleared in xylene, and paraffin-embedded using a tissue processor (Thermo Scientific, Waltham, MA, USA; Excelsior AS) under the overnight protocol. The fully paraffinized kidney tissue was embedded in paraffin and storedat 4 °C. The experimental rats were divided into five groups: (1) physiological saline-infused and injected group (sham) (*n* = 14), (2) E + NE-infused with physiological saline injection (E + NE) (*n* = 13), (3) E + NE-infused with MTP injection (E + NE + MTP) (*n* = 13), (4) E + NE-infused with AT injection (E + NE + AT) (*n* = 14), and (5) E + NE-infused with ICI injection (E + NE + ICI) (*n* = 12). No animals were lost during the experiments.

### 4.3. Biochemical Parameters Detection

Blood was collected via cardiac puncture before rats were sacrificed and transferred to MiniCollect tubes (Greiner Bio-One GmbH, Kremsmünster, Austria; 450533). After centrifugation at 8000 rpm for 10 min at room temperature, serum samples were stored at −80 °C for preservation. The thawed serum was sent to the Department of Pathology and Laboratory Medicine at Kaohsiung Veterans General Hospital for analysis of creatinine and blood urea nitrogen (BUN) concentrations. The concentration of ferrous ions in serum was assayed using the Ferrous Iron Colorimetric Assay kit (Elabscience Biotechnology Inc., Wuhan, China; EBC-K773-M).

### 4.4. Semi-Quantification of Tubulointerstitial Lesion Score and Acute Tubular Necrosis Score

Kidney tissue sections underwent deparaffinization and rehydration before staining with hematoxylin (BioTnA Biotech, Kaohsiung, Taiwan; TA01ES) and eosin (BioTnA Biotech, Kaohsiung, Taiwan; TA01MH) or periodic-acid–Schiff’s (PAS) stains (BioTnA Biotech, Kaohsiung, Taiwan; TASS02-125H). Tissue sectionswere photographed at 200× magnification using a BX51P polarizing microscope (Olympus, Tokyo, Japan). The tubulointerstitial lesion score (TSI) for the cortex and acute tubular necrosis score (ATN) for the OSOM were assessed based on previously described criteria and were semiquantificated by two different researchers and blinded [65].

TSI and ATN were scored on a 0–5 scale by evaluating pathological features, including tubular dilation, atrophy, loss of the brush border, hyaline cast formation, thyroidization, interstitial inflammation, and fibrosis. The judgments of the score were as follows: Grade 0: No pathological areas observed; Grade 1: pathological areas affecting <10% of the field; Grade 2: pathological areas affecting 10–25% of the field;Grade 3: pathological areas affecting 25–50% of the field; Grade 4: pathological areas affecting 50–75% of the field; and grade 5: pathological areas affecting >75% of the field.

The average score from all fields in a tissue section was calculated to obtain the TSI or ATN score, providing a semi-quantitative assessment of renal tubulointerstitial injury.

### 4.5. Iron Ion Detection in Renal Tissue

Deparaffinized and rehydrated kidney tissue sections were stained using a Prussian blue stain kit (BioTnA Biotech, Kaohsiung, Taiwan; TASS15-125) to observe the presence of ferric cations as previously described [66,67,68]. Ferric ions in the tissue sections were detected by reacting acidic potassium ferrocyanide to form Prussian blue [66,68]. To enhance visualization, the Prussian blue signal was amplified using 3,3′-diaminobenzidine of Novolink™ PolymerDetection Systems (Leica Biosystems Nussloch GmbH, Nussloch, Germany; RE7280-K) [66,67]. Tissue sections were photographed at 200× magnification, and the percentage of ferric-cation-positive areas was quantified using ImageJ software version 1.48 (National Institutes of Health, Bethesda, MD, USA). Imaging was performed at ×200 magnification using a BX51P polarizing microscope (Olympus, Tokyo, Japan).

### 4.6. Immunohistochemical (IHC) Staining

Deparaffinized and rehydrated kidney tissue sections underwent antigen retrieval in 0.01 M sodium citrate buffer (pH 6.0) or Epitope Retrieval Solution pH 9 (Leica Biosystems Nussloch GmbH, Nussloch, Germany; RE7119) at 90–100 °C for 20 min, followed by cooling to room temperature. IHC staining was then performed using the Novolink™ Polymer Detection Systems (Leica Biosystems Nussloch GmbH, Nussloch, Germany; RE7280-K) as previously described [9]. The primary antibodies used were as follows: ADRB1 polyclonal antibody (1:200; Bioss; bs-0498R), anti-beta-2 adrenergic receptor antibody [EPR707(N)] (1:200; Abcam, Cambridge, UK; ab182136), Ferritin Heavy Chain Rabbit pAb (1:200; ABclonal, Woburn, MA, USA; A1144), Anti-Lipocalin-2/NGAL antibody (1:400; Abcam; ab63929), and anti-8 hydroxy-2-deoxyguanosine (8-OHdG) antibody (1:50; Santa Cruz, Dallas, TX, USA; sc-66036). Imaging was performed at ×200 magnification using a BX51P polarizing microscope (Olympus, Tokyo, Japan). For statistical analysis, the positive area was quantified using ImageJ software (National Institutes of Health, Bethesda, MD, USA). The protein expression level was quantified as the percentage of the positively stained area [69,70].

### 4.7. Terminal Deoxynucleotidyl Transferase dUTP Nick End Labeling (TUNEL) Assay

Deparaffinized and rehydrated kidney tissue sections were subjected to a TUNEL assay using a commercial kit (MyBioSource, Inc., San Diego, CA, USA; MBS2557027) to evaluate DNA fragmentation. Briefly, sections were treated with proteinase K for 20 min to digest nuclear proteins, followed by labeling of DNA strand breaks with biotin-dUTP using terminal deoxynucleotidyl transferase (TdT) enzyme. TUNEL-positive signals were visualized with 3,3′-diaminobenzidine (DAB) using a biotin–streptavidin–Horseradish peroxidase system. Images were acquired at 200× magnification with a BX51P polarizing microscope (Olympus, Tokyo, Japan). Nuclear damage was quantified by calculating the mean number of positively stained nuclei across all fields for each tissue section.

### 4.8. Protein Extraction and Western Blot Analysis

Proteins were extracted from kidney tissues using a lysis buffer (1:1000, C2978, Sigma-Aldrich, St. Louis, MO, USA) supplemented with a protease inhibitor cocktail (1:1000, HY-K0010, MedChemExpress, Monmouth Junction, NJ, USA), phosphatase inhibitor cocktail I (1:1000; HY-K0021, MedChemExpress, Monmouth Junction, NJ, USA), and phosphatase inhibitor cocktail II (1:1000, HY-K0022, MedChemExpress, Monmouth Junction, NJ, USA). Protein concentrations were determined using the Bradford protein assay (Coomassie Plus protein assay reagent, Thermo Fisher Scientific, Waltham, MA, USA).

The following primary antibodies were used: Phospho-p38 MAPK antibody (Thr180/Tyr182) (1:1000, 9211, Cell Signaling Technology, Danvers, MA, USA), p38 MAPK antibody (1:1000, #9212, Cell Signaling, Danvers, MA, USA Technology), ACSL4 antibody (F-4) (sc-365230, Santa Cruz Biotechnology, Dallas, TX, USA), GPX4 antibody (67763-1-Ig, Proteintech Group, Inc., Rosemont, IL, USA), E-Cadherin Rabbit mAb (1:1000,A20798, ABclonal Inc.), N-Cadherin antibody (1:1000,A19083, ABclonal Inc.), Klotho antibody (1:1000,28100-1-AP, Proteintech Group, Inc., Rosemont, IL, USA), ALOX12(1:1000,A14703, ABclonal Inc.), ALOX15(1:1000,TA504250S, Origene Technologies Inc., Rockville, MD, USA) and β-actin antibody (1:1000,66009-1-Ig, Proteintech Group, Inc., Rosemont, IL, USA).

Protein detection was conducted using the SuperSignal™ Western Blot Substrate Bundle (A45917, Thermo Fisher Scientific, Waltham, MA, USA). Images were captured using the ChemiDoc™MP Imaging System (Bio-Rad Laboratories, Inc., Hercules, CA, USA) and analyzed with Image Lab software (version 6.0, Bio-Rad). Data were normalized to β-actin levels and expressed as a percentage change relative to the sham group.

### 4.9. Renal Vessel Morphology

The wall-to-lumen area ratio of the renal arcuate arteries was calculated using elastica van Gieson (EVG) staining [71,72]. The vascular lesion score was assessed based on PAS staining following the scoring protocol described in a previous study [73]. Detailed procedures are provided in the Supplementary Materials and Methods.

### 4.10. Statistical Analysis

All results were calculated using a nonparametric Kruskal–Wallis H test, followed by a Mann–Whitney U-test. All statistical graphs are presented as mean ± standard deviation (mean ± SD). A *p*-value of <0.05 was considered statistically significant. Statistical analyses were performed using IBM SPSS Statistics Version 20 software (IBM Corp., Armonk, NY, USA, 2011) and GraphPad Prism version 6.5 for Windows (GraphPad Software Inc., San Diego, CA, USA).

## 5. Conclusions

In conclusion, our research provides innovative insights into the connection between catecholamine storms, β-adrenoceptors, and ferroptosis in AKI. Excess catecholamines activate β1-adrenoceptors, leading to acute renal tubular injury. MTP, a lipophilic β1-adrenoceptor blocker metabolized by hepatic P450-associated enzymes, demonstrates superior therapeutic efficacy in suppressing apoptosis, oxidative stress and ferroptosis, thereby improving both pathological and biochemical disorders in catecholamine-induced AKI. While β2-adrenoceptor blockers can alleviate some pathological injury, they do not reverse renal dysfunction, suggesting a more complex role for β2-adrenoceptors in the mechanism of kidney injury during catecholamine overdose. Our findings highlight the therapeutic potential of MTP in reducing the risk of AKI caused by excess catecholamine exposure in patients.

## Data Availability

The original contributions presented in this study are included in the article. Further inquiries can be directed to the corresponding author.

## Supplementary Materials

The following supporting information can be downloaded at: https://www.mdpi.com/article/10.3390/ijms27125480/s1.

## Abbreviations

The following abbreviations are used in this manuscript:

8-OHdG	8-hydroxy-2′-deoxyguanosine
ALOX12	arachidonate 12-lipoxygenase
ALOX15	arachidonate 15-lipoxygenase
ACSL4	acyl-CoA synthetase long chain family member 4
ADRB1	β1-adrenoceptor
ADRB2	β2-adrenoceptor
AKI	acute kidney injury
AT	atenolol
ATN	acute tubular necrosis
BUN	3,3′-diaminobenzidine
E	epinephrine
ELISA	enzyme-linked immunosorbent assay
EVG	Elastica-van Gieson staining (Weigert’s method)
FtH	ferritin heavy chain
GPX4	glutathione peroxidase type 4
H&E	hematoxylin and eosin
ICI	ICI 118,551
IHC	immunohistochemistry
MAPK	mitogen-activated protein kinase
MATE	multidrug and toxin extrusion protein
MTP	metoprolol
NE	norepinephrine
NGAL	neutrophil gelatinase-associated lipocalin
OCT2	organic cation transporter 2
OSOM	outer stripe of the outer medulla
PAS	Periodic-Acid–Schiff staining
p-p38	phosphorylated p38
PUFA	polyunsaturated fatty acid
SD	standard deviation
TdT	terminal deoxynucleotidyl transferase
TSI	tubulointerstitial injury score
TUNEL	terminal deoxynucleotidyl transferase dUTP nick-end labeling
