# Supplementary material for: Protective Effects of Selective β-Adrenoceptor Blockade on Renal Pathophysiology in a Catecholamine Storm of Rat
Source: Int J Mol Sci. 2026 Jun 17;27(12):5480. doi: 10.3390/ijms27125480 (PMC13299829; doi:10.3390/ijms27125480)
Supplement: Supplementary file 1 [file ijms-27-05480-s001.zip › manuscript_Supplementary document_1st.pdf]

1. Supplementary Material and Method

1.1. Renal Vessel Morphology

Kidney tissue sections were incubated in Bouin's solution (Sigma-Aldrich, HT10132) at 56°C after deparaffinization and dehydration. The sections were then stained with elastica van Gieson (EVG) according to Weigert's staining protocol (Merck Millipore, 1.15974.0002) [1]. Arcuate arteries observed in the renal tissue were photographed at 200x magnification. Imaging was performed at 200× magnification using a BX51P polarizing microscope (Olympus, Tokyo, Japan). The area fraction of the arterial wall to the lumen was measured using ImageJ software to calculate the wall-to-lumen ratio, which was used to assess vascular remodeling in the kidneys [2].

1.2. Vessel Lesion Score

The arcuate artery stained with periodic acid-Schiff's (PAS) staining was semi-quantified using a vessel lesion score, as described in reference [3]. Briefly, the scoring criteria were as follows: Grade 0: the wall-to-lumen diameter ratio of the arcuate artery is greater than 0.6; Grade 1: the wall-to-lumen diameter ratio is less than 0.6 but greater than 0.5; Grade 2: the wall-to-lumen diameter ratio is less than 0.5 but greater than 0.2; and Grade 3: the wall-to-lumen diameter ratio is less than 0.2 but without complete occlusion. The average score from all fields in a tissue section was calculated to obtain the vessel lesion score, providing a semi-quantitative assessment of arterial injury. Arcuate arteries observed in the renal tissue were photographed at 200x magnification. Imaging was performed at 200× magnification using a BX51P polarizing microscope (Olympus, Tokyo, Japan).

The average score from all fields in a tissue section was calculated to obtain the vessel lesion score, providing a semi-quantitative assessment of artery injury.

1.3. *Statistical Analysis*

All results were calculated using a nonparametric Kruskal-Wallis H test, followed by a Mann-Whitney U-test. All statistical graphs are presented as mean ± standard deviation (mean ± SD). A p value of <0.05 was considered statistically significant. Statistical analyses were performed using IBM SPSS Statistics Version 20 software (IBM Corp., Armonk, NY, USA, 2011) and GraphPad Prism version 6.5 for Windows (GraphPad Software. Inc., San Diego, CA, USA www.graphpad.com).

2. Supplementary Figure

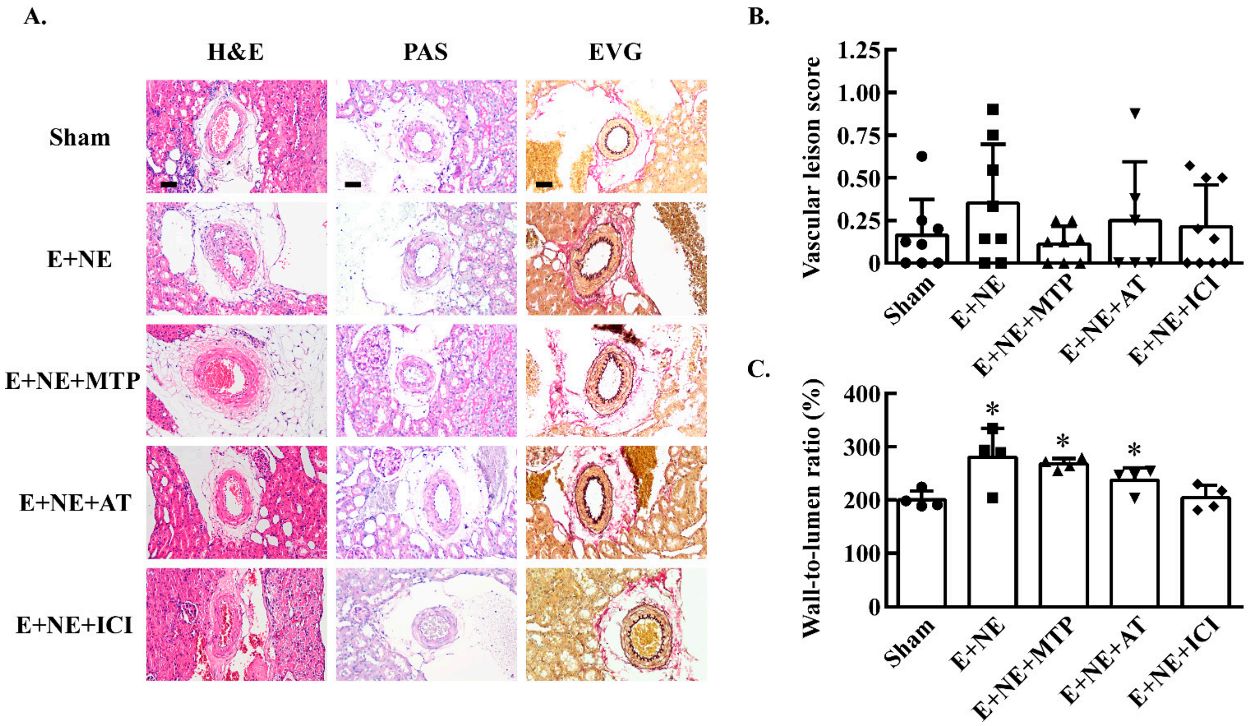

Figure S1. Morphological changes of arcuate vessels in the cortex and OSOM following β-adrenoceptor blocker interventions in a catecholamine overload rodent model. A. The morphological appearance of arcuate arteries was observed using H&E or PAS. EVG staining was used to assess structural changes in the vessel wall and lumen. Images were captured at 200× magnification with a scale bar of 50 μm. **B.** Vascular lesion scores were semi-quantitated from PAS staining in arcuate arteries of kidney. Sample size: n = 6 for all groups. **C.** The wall-to-lumen ratio was measured using EVG staining in arcuate arteries. Sample size: n = 4 for all groups. Data are presented as means ± SD. Statistical analyses were performed using the Kruskal-Wallis H test and post hoc Mann-Whitney U test. *, $p < 0.05$ vs. sham; #, $p < 0.05$ vs. E+NE. AT: atenolol, E: epinephrine, EVG: Elastica-van Gieson staining (Weigert's method), H&E: Hematoxylin and Eosin staining, ICI: ICI 118,551, MTP: metoprolol, NE: norepinephrine, OSOM: outer stripe of the outer medulla, PAS: Periodic Acid-Schiff staining.

Supplementary References

1. Hernandez-Morera, P.; Travieso-Gonzalez, C.M.; Castano-Gonzalez, I.; Mompeo-Corredera, B.; Ortega-Santana, F. Segmentation of elastic fibres in images of vessel wall sections stained with Weigert's resorcin-fuchsin. *Comput Methods Programs Biomed* **2017**, *142*, 43-54, doi:10.1016/j.cmpb.2017.02.018.
2. Wu, C.J.; Li, Y.H.; Wu, F.Z.; Chen, H.H. Eplerenone improves hyperglycemia and sympathetic excitation in chronic renocardiac syndrome in rats. *Naunyn Schmiedebergs Arch Pharmacol* **2024**, *397*, 1081-1092, doi:10.1007/s00210-023-02665-5.
3. Miyaoka, Y.; Okada, T.; Tomiyama, H.; Morikawa, A.; Rinno, S.; Kato, M.; Tsujimoto, R.; Suzuki, R.; China, R.; Nagai, M.; et al. Structural changes in renal arterioles are closely associated with central hemodynamic parameters in patients with renal disease. *Hypertens Res* **2021**, *44*, 1113-1121, doi:10.1038/s41440-021-00656-8.
